# The Early Facilitative and Late Contextual Specific Effect of the Color Red on Attentional Processing

**DOI:** 10.3389/fnhum.2018.00224

**Published:** 2018-06-11

**Authors:** Tao Xia, Zhengyang Qi, Jiaxin Shi, Mingming Zhang, Wenbo Luo

**Affiliations:** ^1^Research Center of Brain and Cognitive Neuroscience, Liaoning Normal University, Dalian, China; ^2^Department of Psychology, The University of Hong Kong, Hong Kong, Hong Kong

**Keywords:** red-angry, green-happy, attention bias, Color-in-Context theory, ERPs

## Abstract

Many studies have proved that color represents a variety of emotionally meaningful information. Researchers have proposed that context information endows colors with different associated meanings, and elicits corresponding behavior. Others have contended that the color red intensifies the stimulus’ existing valence or motivation tendency in the early processing step. The present study attempts to incorporate these two effects of the color red to explore their differences in a dot probe task, using event-related potential (ERP). Our ERP results indicate that the color red intensifies the initial attention to emotion-congruent conditions, as indicated by the P1 component. However, the colors red and green lead to sustained attention to the expression of anger and happiness, respectively, but not fear, as shown by the late positive complex component (all results are available at: https://osf.io/k3b8c/). This study found the different processing stages of the effect of the color red during attentional processing in a discrete emotional context, using ERPs, and may refine the Color-in-Context theory.

## Introduction

As a basic dimension of human perception, color is ubiquitous in our surroundings, and plays a fundamental role in human perception and experience of the world (Valdez and Mehrabian, 1994; Müller et al., 2006; Bramão et al., 2011). Researchers have observed that the same color can convey inconsistent meanings under different conditions. For instance, the color red is not only associated with negative meanings but also linked to positive meanings in both natural and human societies. Specifically, in natural environments, the color red often plays an important role in warning the body of potential hazard from insects, birds, or reptiles (Stevens and Ruxton, 2012). On the other hand, the same color could also be an indication of ripe fruits which attract animals to consume them for living. In our daily life, the color red is often used to indicate dangerous situations which, if not avoided, will result in injury. Meanwhile, it is also a symbol of luck, festivities, and other positive themes in some cultures, such as in Chinese culture. The fact that the same color may convey contrary meanings in different situations is of scientific interest to investigators who wish to study its impact on the individual’s psychological functioning.

Since the color red is associated with both negative and positive meanings, scholars have proposed the *Color-in-Context* theory, which states that the context in which the color red is perceived, influences people’s interpretation of its associated meaning, and subsequently alters their behaviors accordingly (Elliot and Maier, 2012, 2014; Elliot, 2015). The *Color-in-Context* theory hypothesizes that colors convey different meanings depending on the context (Elliot and Maier, 2012). Some studies have found that the color red, associated with danger and generally negative meanings in an achievement context, activates withdrawal responses, and influences cognition, emotion, and behavior (Moller et al., 2009; Kuhbandner and Pekrun, 2013; Pravossoudovitch et al., 2014; Shi et al., 2015). In contrast to achievement contexts, studies indicate that, in romantic contexts, the color red is associated with sexual attractiveness, and activates approach motivation, and impacts mating behavior in heterosexual individuals (Elliot and Niesta, 2008). However, recently, researchers have failed to replicate this attractive effect (Peperkoorn et al., 2016). In addition to being associated with danger or sexual desirability in different contexts, the color red is also an inherent feature of angry expressions in an emotional context. When angry, the faces of humans and other primates often turn red (Drummond, 1997; Changizi et al., 2006). The color red, in contrast to blue and gray, thus, facilitates the identification of angry, but not fearful, expressions which suggests a more specific association between the color red and anger (Young et al., 2013).

In addition to highlighting the important role of context information in color effect, researchers have recently suggested that attention may be involved in the context-dependency of the associations of the color red (Buechner et al., 2014, 2015; Buechner and Maier, 2016). An attentional-bias theory has been proposed, which hypothesizes that the color red could led to an automatic attentional bias toward stimuli that existing attentional priority caused by motivation tendency, making the target more prominent than others. Buechner et al. (2014) proposed that the color red intensifies the stimuli’s existing valence or motivation tendency and impact on human behaviors in early processing steps. In a modified dot probe task, the reaction times show that the color red intensifies the perceiver’s attentional engagement to angry and happy, but not neutral, expressions, in contrast to blue. Using a dot probe task with pictures of emotional scenes from International Affective Picture System (IAPS), studies of ERP components [early directing attention negativity (EDAN) and anterior directing attention negativity (ADAN)] have also revealed that the color red captures initial and later attention in both positive and negative conditions, but not in a neutral condition (Kuniecki et al., 2015). This result is consistent with Buechner’s proposal that the color red intensifies the stimuli’s existing motivation tendency (emotion effect). However, it is only involved in the valence of the stimuli, and does not influence more specific associations (e.g., anger) in attentional processing.

Taken together, previous studies have shown that the color red intensifies the stimulus’ existing valence or motivation information in early processing steps. Context-specific effects may then emerge and influence human behavior (Buechner et al., 2014, 2015). It is important to investigate the relationship between attentional bias and context-specific information during visual processing in the presence of the color red, as it is useful to refine the theory of the influence of color on psychological functioning.

In this study, we attempted to integrate the two effects of the color red, and investigate its differential effects on attentional processing using ERPs, as it is an excellent tool to study the time course of mental processes. We hypothesized that the existing emotional attention aspects of red stimuli captures the perceiver’s attentional resources in an early processing stage, while context information regarding discrete emotional association sustains the individual’s attention to corresponding red targets in the late processing stage. A modified dot probe task, whose original version is often used to measure the attentional bias of emotional stimuli, has been used in this study, although we changed the target colors to red and green. Angry, fearful, and happy expressions were used as cues to create an emotional context. We selected the color green as a control color, as this condition has been used successfully in several previous studies (Elliot et al., 2007; Maier et al., 2009). Indeed, some researchers contend that green is a pleasant hue, and enhances the recognition of happy expressions. In fact, even in a cycling task, the color green makes individuals feel happy (Valdez and Mehrabian, 1994; Akers et al., 2012; Gil and Le Bigot, 2014). In previous studies, it has been shown that the P1 component of ERP is a good marker for capturing initial attention in the dot probe task (Brosch et al., 2008; Liu et al., 2013). The participants may also direct their attention to relevant stimuli and perform elaborate processing, as evidenced by a large late positive complex (LPC) in ERP (Jaworska et al., 2012; Gable and Adams, 2013; Zhang et al., 2014; Yi et al., 2015; Zhu et al., 2015). Therefore, we predicted that the color red may capture the initial attention to attended stimuli for all emotions (anger, fear, and happiness), as it leads to larger P1 responses. However, in the late processing stage, only angry facial expressions sustained the perceiver’s attention to the red stimuli, as evidenced by a larger LPC.

## Materials and Methods

### Participants

We did not run a power analysis to estimate our sample size before the study. And we decided the sample size based on our previous study (Liu et al., 2013; Zhang et al., 2014; Zhu et al., 2015). Seventy-two undergraduates were recruited from Chongqing University of arts and sciences in exchange for payment. They were randomly assigned: behavioral experiment (*n* = 31, 20 females, mean = 22, *SD* = 1.67) and ERP experiment (*n* = 41, 30 females, mean = 21.5, *SD* = 1.96). All participants were reported right-handed and having normal or corrected-to-normal vision without any color deficiencies. All subjects were provided informed written consent prior to the study. The study was approved by Chongqing University of Arts and Sciences Human Research Institutional Review Board in accordance with the Declaration of Helsinki (1991).

### Stimuli

Sixty faces (10 angry, 10 happy, 10 fear, and 30 neutral faces) were chosen from the Chinese Facial Affective Picture System (Gong et al., 2011) depicting the emotion of people in black and white photograph, with an equal number of face pictures of males and females. We also assessed the valence and arousal on a 9-point scale with a sample of 45 Chinese subjects. We analyzed the average score of all stimuli in our experiments which are reported in **Table 1**. The statistical results showed that angry and fearful faces were not significantly different in emotional valence [*F*(3,44) = 203.09, *p* < 0.001, η^2^ = 0.93, 95% CI (0.88, 0.95); anger vs. fear: *p* = 0.91], while their valences were significantly different from happy faces (*p*s < 0.001) and neutral faces (*p*s < 0.001). The average arousal score between angry, fearful, and happy faces was not significantly different from each other [*F*(3,44) = 53.54, *p* < 0.001, η^2^ = 0.79, 95% CI (0.64, 0.84); anger vs. fear vs. happiness: *p*s > 0.10], while they were significantly different from neutral faces (*p*s < 0.01). In addition, each facial expression had also been assessing the recognition rates and had been used successfully in previous studies.

**Table 1:** Average ratings (mean ± SD) for valence and arousal of stimuli.

	Arousal	Valence
Anger	6.34 ± 1.20	2.82 ± 0.44
Fear	6.23 ± 1.68	2.81 ± 0.44
Happiness	5.97 ± 0.98	5.74 ± 0.89
Neutral	3.63 ± 0.54	4.12 ± 0.70

*45 participants assessed the valence and arousal of sixty faces (10 angry, 10 happy, 10 fear, and 30 neutral faces) on a 9-point scale, and we analyzed the average scores of all stimuli per condition in our experiments*.

Stimuli (260 pixels × 300 pixels) were presented on a liquid crystal display monitor (17-inch) at a viewing distance of 100 cm. The viewing angle was 3.9° × 4.5°, and the screen resolution was 72 pixels per inch.

### Procedure

**FIGURE 1:**
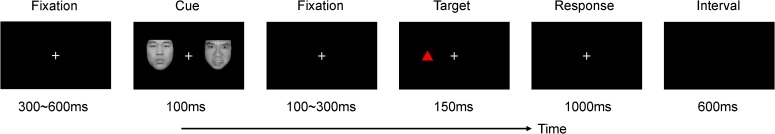
Schematic representation of experimental procedure. Each trial contained a cue (an angry/fear/happy face in one side of the screen and a neutral face in the other side) and a target stimulus (a red/green triangle that might be upper or lower). Congruent (the emotional face and the target appeared at the same position) and incongruent (the emotional face and the target appeared at different positions) trials were appeared in random order with equal probability (50% each). Participants were required to judge the target position by pressing “f” (left) or “j” (right), and they were instructed to respond only one kind of triangle (the upper or the lower, counterbalanced across subjects) but ignore its color. Importantly, we used a different percent of responding trials in our EEG (go trials, 10%, no-go trials, 90%) and behavioral tasks (go trials, 50%, no-go trials, 50%) to study the spatial orienting in EEG task.

The experiment is within subject design and consisted of one practice block of 12 trials, followed by one experimental block of 960 trials. The participants had a rest period of 1 min every 160 trials. All trials were randomized. An example of the stimuli and the trial design of the experiment is illustrated in **Figure 1**. There were three factors in our experiment, including emotion (anger, fear, happy), congruency (congruent, incongruent), and color (red and green). A fixation cross appeared in the center of the screen for 300 to 600 ms, and was followed by a cue that consisted of two faces. There was an emotional (angry, fear, or happy) and a neutral face on the left or right side of the screen. After a short interval (100 ms to 300 ms), a target appeared at either the same position as the emotional face (congruent) or at a different position (incongruent) for 150 ms. Congruent and incongruent trials appeared in random order with equal probability (50% each). To match the lightness we used the BabelColor Translator and Analyzer (CT&A) to transform the parameters of target colors from Adobe RGB (1998) color space (red = 255, green = 0, blue = 0; red = 0, green = 181, blue = 0) into CIE LCh color space (red: L = 61.4, C = 117, h = 40.0; green: L = 61.2, C = 128, h = 147). After the appearance of the target, the participants had to assess the position of the target as quickly and as accurately as possible. If the triangle was presented on the left, targets had to press “F” on the computer keyboard using their left index fingers. Otherwise, they were to press “J” using their right index fingers. The target was one of four types of triangle (red upper, red lower, green upper, and green lower). The participants were instructed to respond to only two types of triangles (red and green upper, or red and green lower, counterbalanced across subjects), but to ignore its color. Before the next trial started, the participants had a maximum of 1,000 ms to respond. Importantly, we used different percentages of response trials in our behavioral tasks (go trials, 50%; no-go trials, 50%) and electroencephalogram (EEG) studies (go trials, 10%; no-go trials, 90%) in order to study spatial orientation in the EEG task (Brosch et al., 2008; Liu et al., 2013). In addition, since we analyzed no-go, not go, trials in our ERP analysis, the ERPs (P1, LPC) may not be related to the behavioral response. The behavioral experiment is only for repeating the similar results of the previous study. Our main interests are focused on the ERPs results since we want to clarify the different stage of the color red and its effect on attentional processing.

### EEG Recording and Analysis

Brain electrical activity was recorded at 64 scalp sites using tin electrodes mounted in an elastic cap with a sampling frequency of 500 Hz (Brain Products, Munich, Germany), according to the international 10–20 System. FCz was used as the reference, and ground electrode was on the medial frontal aspect. The horizontal EOG was recorded from the right orbital rim. All electrode impedance was <5 kΩ. The EEG and EOG were amplified using a 0.01–100 Hz bandpass.

EEG data were analyzed using BrainVision Analyzer (2.1) software (BrainProducts GmbH). Data were off-line mathematically re-referenced to the left and right mastoids, and filtered with band pass filter 0.1–30 Hz (24 dB). Filtered data were segmented beginning 100 ms prior to the onset of the target stimulus array and lasting for 950 ms. An ocular artifact reduction procedure (Semlitsch et al., 1986) based on right eye HEOG activity was used to remove blink artifacts. Baseline correction was performed using 100 ms prestimulus interval. EEG epochs in which the signal exceeded ± 100 μV were excluded. Artifact-free epochs were averaged separately for each electrode, condition, and individual. The average ERPs of the 41 subjects were computed based on no-go trials (80 ^∗^ 90% = 72 trials precondition).

We analyzed the amplitudes of occipital P1 and LPC components across different set of electrodes in line with grand-mean ERP topographies and previous literatures (Yi et al., 2015), the mean amplitude of P1 was calculated at the electrode sites of PO3, PO4, PO7 and PO8 (time window = 100–150 ms). The mean amplitude of LPC was calculated at electrode sites C3, C4, Cz, CP3, CP4, CPz (time window = 340–460 ms). For each component, a four-way repeated-measures ANOVA was performed with the following variables as within-subject factors: “Color” (Red target vs. Green target), “Congruency” (Congruent vs. Incongruent), “Emotion” (Anger vs. Fear vs. Happy), and “Hemisphere” (P1: Left hemisphere vs. Right hemisphere; LPC: Left hemisphere vs. Medal region vs. Right hemisphere). *P* value was corrected using the Greenhouse–Geisser method.

## Results

Means and standard error (mean ± SE) of behavioral data at four experimental conditions are reported in **Table 2**, and more details are reported in **Table 3** with means and standard deviation (mean ± SD). Means and standard errors (mean ± SE) of electrophysiological data at different experimental conditions are reported in **Table 4**.

**Table 2:** Reaction time (mean ± SE) at four experimental conditions of Behavioral experiment (*N* = 31) and ERP experiment (*N* = 41).

	Behavioral experiment	ERP experiment
Congruent-Red (ms)	375 ± 16	308 ± 11
Congruent-Green (ms)	357 ± 15	292 ± 11
Incongruent-Red (ms)	366 ± 14	308 ± 10
Incongruent-Green (ms)	364 ± 15	295 ± 11

**Table 3:** Reaction time (mean ± SD) at all experimental conditions.

	Anger				Fear				Happiness			
	Congruent		Incongruent		Congruent		Incongruent		Congruent		Incongruent	
	Red	Green	Red	Green	Red	Green	Red	Green	Red	Green	Red	Green
Behavioral exp. (ms)	375 ± 96	358 ± 92	367 ± 86	355 ± 76	374 ± 97	350 ± 79	367 ± 78	377 ± 96	375 ± 92	363 ± 100	365 ± 77	362 ± 96
ERP exp. (ms)	309 ± 80	293 ± 73	307 ± 64	290 ± 66	307 ± 70	295 ± 74	313 ± 75	299 ± 73	309 ± 70	288 ± 72	306 ± 72	295 ± 84

**Table 4:** Amplitudes (mean ± SE) of P1 and LPC at all experimental conditions.

	Anger				Fear				Happiness			
	Congruent		Incongruent		Congruent		Incongruent		Congruent		Incongruent	
	Red	Green	Red	Green	Red	Green	Red	Green	Red	Green	Red	Green
P1 (μV)	0.83 ± 0.32	0.38 ± 0.27	0.72 ± 0.32	0.50 ± 0.27	0.86 ± 0.30	0.39 ± 0.31	0.50 ± 0.28	0.53 ± 0.27	0.93 ± 0.29	0.54 ± 0.29	0.72 ± 0.27	0.40 ± 0.33
LPc (μV)	7.96 ± 0.67	7.00 ± 0.61	7.63 ± 0.67	7.20 ± 0.58	7.07 ± 0.63	7.20 ± 0.64	7.24 ± 0.67	7.19 ± 0.53	6.77 ± 0.58	7.27 ± 0.57	6.84 ± 0.57	7.21 ± 0.60

### Behavioral Results

We only analyzed the reaction time in both EEG and behavioral experiment because there was a ceiling effect in accuracy as the task is too simple. In the behavioral experiment, the main effect of “Color” [*F*(1,30) = 5.34, *p* = 0.03, η^2^ = 0.15, 95% CI (0,0.37)] was significant. Participants performed slower in red triangle (371 ± 15 ms) than green triangle (361 ± 15 ms). However, the main effects of “Emotion” and “Congruency” [*Fs* < 0.45, *p*s > 0.63] were not significant. There was a significant interaction between “Congruency” and “Color” [F(1,30) = 5.02, *p* = 0.03, η^2^ = 0.14, 95% CI(0, 0.36)]. Simple effects analysis demonstrated that the reaction time of “Congruency” was significantly influenced by “Color”. Participants performed slower to red triangle than green triangle in congruent condition (375 ± 16 ms vs. 357 ± 15 ms, *t*(30) = 3.77, *p* < 0.001, *d* = 0.68, 95% CI (0.28, 1.06)); however, there was no difference between red triangle and green triangle in incongruent condition (366 ± 14 ms vs. 365 ± 15 ms, *t*(30) = 0.26, *p* = 0.80). And there were no other significant interactions among “Color”, “Emotion” and “Congruency” [*Fs* < 1.82, *p*s > 0.18].

In the EEG experiment, the main effect of “Color” [*F*(1,40) = 31.89, *p* < 0.001, η^2^ = 0.44, 95% CI(0.21, 0.60)] was significant. Participants performed slower to red triangle (308 ± 10 ms) than green triangle (293 ± 11 ms). While the main effect of “Emotion” and “Congruency” [*Fs* < 1.24, *p*s > 0.30], as well as all their interactions [*Fs* < 0.85, *p*s > 0.43] were not significant.

### P1 Component

**FIGURE 2:**
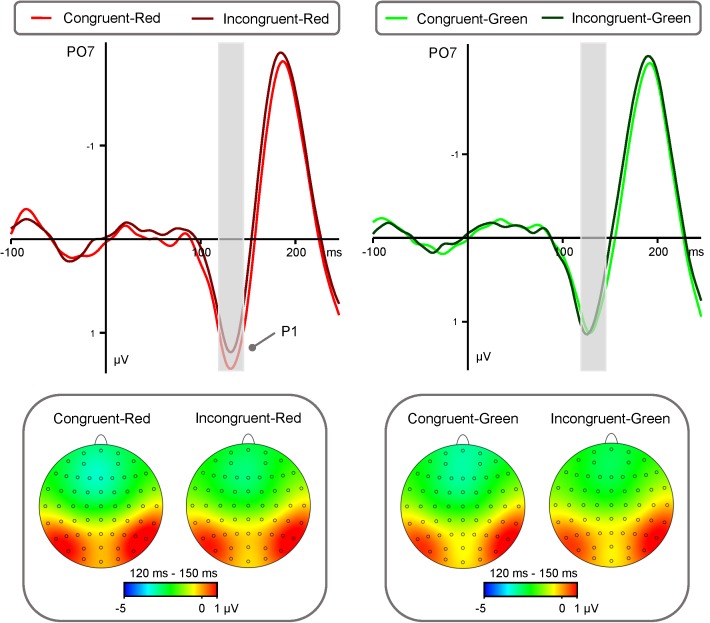
Group-level average ERP waveforms and scalp topographies for the interaction between the effects “Congruency” and “Color” of P1. ERP waveforms of P1 component (recorded at electrodes PO3, PO4, PO7, PO8) are shown for Congruent-Red target (red lines), Incongruent-Red target (dark red lines), Congruent-Green target (green lines), and Incongruent-Green target (dark green lines). Left panel: ERP waveforms and scalp topographies of P1 component generated by Congruent-Red and Incongruent-Red conditions. Right panel: ERP waveforms and scalp topographies of P1 component generated by Congruent-Green and Incongruent- Green conditions.

P1 amplitude (**Figure 2**) showed a significant main effect of “Color” [*F*(1,40) = 17.25, *p* < 0.001, η2 = 0.30, 95 % CI (0.08, 0.49)]. Red triangle (0.76 ± 0.28 μV) elicited significantly larger P1 amplitude than green triangle (0.44 ± 0.27 μV). While the main effects of “Emotion”, “Congruency” and “Hemisphere” were not significant [*Fs* < 1.77, *p*s > 0.19].

In addition, there was a significant interaction between the effects “Congruency” and “Color” [*F*(1,40) = 7.15, *p* = 0.011, η^2^ = 0.15, 95% CI (0.01, 0.35)]. Simple effects analysis indicated that the effect of “Color” significantly influenced the amplitudes of “Congruency”. Simple effects analysis showed that congruent condition elicited significantly larger P1 amplitude than incongruent condition [0.87 ± 0.28 μV vs. 0.65 ± 0.28 μV, *t*(40) = 3.01, *p* < 0.01, *d* = 0.47, 95% CI (0.15, 0.79)] in the red condition. While there was no difference between congruent condition and incongruent condition (0.44 ± 0.27 μV vs. 0.45 ± 0.27 μV, *t*(40) = -0.15 *p* = 0.88) in the green condition.

### LPC Component

**FIGURE 3:**
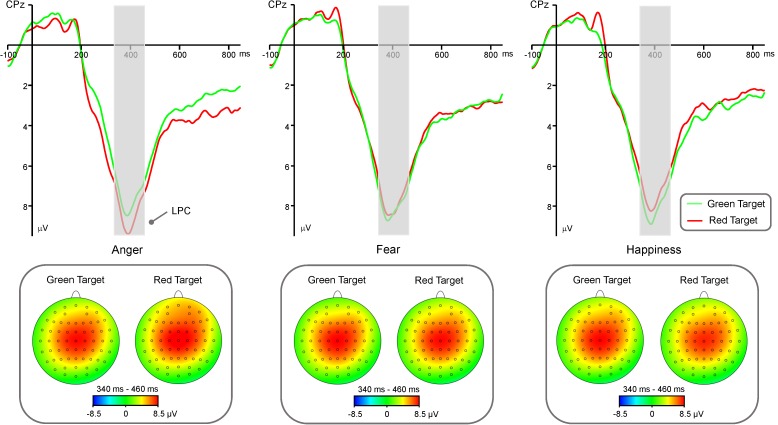
Group-level average ERP waveforms and scalp topographies for Anger, Fear, and Happiness conditions. ERP waveforms of LPC component are shown for red target (red lines) and green target (green lines), and average LPC waveforms recorded at electrodes C3, C4, Cz, CP3, CP4, CPz. Left panel: ERP waveforms and scalp topographies of LPC component generated by anger faces. Mid-line panel: ERP waveforms and scalp topographies of LPC component generated by fear faces. Right panel: ERP waveforms and scalp topographies of LPC component generated by happy faces.

LPC amplitude (**Figure 3**) showed significant main effects of “Emotion” [*F*(2,40) = 4.19, *p* = 0.02, η^2^ = 0.17, 95% CI (0, 0.35)] and “Hemisphere” [*F*(2,40) = 24.89, *p* < 0.001, η^2^ = 0.55, 95% CI (0.31, 0.68)]. *Post hoc* pairwise comparisons showed that anger faces elicited significantly larger LPC amplitude than happy faces (7.44 ± 0.61 μV vs. 7.02 ± 0.59 μV, *p* = 0.01), while there was no difference between fearful faces and happy faces (7.17 ± 0.59 μV vs. 7.02 ± 0.59 μV, *p* = 0.24) or between fearful faces and angry faces (7.17 ± 0.59 μV vs. 7.44 ± 0.61 μV, *p* = 0.09). In addition, LPC amplitude was significantly larger in the mid-line region (8.27 ± 0.67 μV) than in the left hemisphere (6.65 ± 0.58 μV, *p* < 0.001) and right hemisphere (6.72 ± 0.58 μV, *p* < 0.001), while there was no difference between left hemisphere and right hemisphere (*p* = 0.80). The main effects of “Color” and “Congruency” were not significant [*Fs* < 0.36, *p*s > 0.55].

There was a significant interaction between the effects “Emotion” and “Color” [*F*(1, 40) = 9.35, *p* < 0.001, η^2^ = 0.19, 95% CI (0.02, 0.38)]. Simple effects analysis showed that red triangle elicited significantly larger LPC amplitude than green triangle in angry condition [7.80 ± 0.66 μV vs. 7.09 ± 0.58 μV, *t*(40) = 3.23, *p* = 0.002, *d* = 0.51, 95% CI (0.18, 0.83)], while red triangle elicited significantly smaller than green triangle in happy condition [6.81 ± 0.56 μV vs. 7.24 ± 0.58 μV, *t*(40) = -2.27, *p* = 0.029, *d* = -0.35, 95% CI (-0.67, -0.04)], and there was no difference between red triangle and green triangle in fear condition (7.15 ± 0.64 μV vs. 7.20 ± 0.56 μV, *t*(40) = -0.23, *p* = 0.82). In addition, there was a significant interaction of “Emotion,” “Color,” and “Hemisphere” [*F*(1,40) = 6.01, *p* < 0.001, η^2^ = 0.13, 95% CI(0,0.32)]. Simple effects analysis showed that red triangle under angry condition elicited significantly larger LPC amplitude than green triangle under angry condition in the left hemisphere [7.30 ± 0.62 μV vs. 6.46 ± 0.54 μV, *t*(40) = 3.92, *p* < 0.001, *d* = 0.61, 95% CI (0.27, 0.94)] and mid-line region [8.88 ± 0.76 μV vs. 8.06 ± 0.68 μV, *t*(40) = 3.32, *p* = 0.02, *d* = 0.52, 95% CI(0.19, 0.84)], and red triangle under angry condition elicited marginally larger than green triangle under angry condition in the right hemisphere [7.21 ± 0.66 μV vs. 6.76 ± 0.59 μV, *t*(40) = 1.96, *p* = 0.06, *d* = 0.31, 95% CI (-0.01, 0.62)]. There was no difference between red triangle and green triangle under fearful condition in the left hemisphere (6.59 ± 0.59 μV vs. 6.62 ± 0.54 μV, *t*(40) = -0.18, *p* = 0.86), mid-line region (8.19 ± 0.74 μV vs. 6.46 ± 0.54 μV, *t*(40) = -0.81, *p* = 0.42), or right hemisphere (6.69 ± 0.62 μV vs. 6.60 ± 0.57 μV, *t*(40) = 0.46, *p* = 0.65). Green triangle under happy condition elicited significantly larger LPC amplitude than red triangle under happy condition in the left hemisphere [6.65 ± 0.54 μV vs. 6.26 ± 0.53 μV, *t*(40) = -2.08, *p* = 0.044, *d* = -0.33, 95% CI (-0.64, -0.01)] and mid-line region [8.36 ± 0.66 μV vs. 7.80 ± 0.65 μV, *t*(40) = -2.52, *p* = 0.016, *d* = -0.39, 95% CI (-0.71, -0.07)], while there was no difference between green triangle and red triangle under happy condition in the right hemisphere (6.70 ± 0.59 μV vs. 6.37 ± 0.55 μV, *t*(40) = -1.76, *p* = 0.09).

## Discussion

In this study, we used behavioral measures and ERPs to assess the relationship between context information and attentional bias to the color red during visual processing. In a discrete emotional context, our behavioral results indicated that the reaction time for the color red was longer than that for color green. Moreover, the reaction time for the color red in the congruent condition was longer than that for the color green. The ERP results indicate that the color red captures initial attention in the congruent condition while the valence is ignored, as shown by the P1 component. We also found that the colors red and green led to sustained attention to angry and happy faces in the late processing stage in congruent and incongruent conditions, respectively, as determined using the LPC component. In addition, we reported the eta-squared, not partial eta-squared, and Cohen’s d values and their 95% confidence interval in the current study. The eta-squared values range from 0.13 to 0.55 and the absolute d values range from 0.33 to 0.68 among the statistically significant results, which implies a moderate strength of association, representing effective experimental control, among experiment factors, reaction time, and ERPs (Pierce et al., 2004). Additionally, the effect size used in this study was similar to that used in previous related studies which indicated that the color red is a factor that influences an individual’s psychological functioning (Buechner et al., 2014; Kuniecki et al., 2015).

It is noteworthy that both in the behavioral and electroencephalography (EEG) experiments conducted previously, there were no main effects of congruency and the interaction of emotion and congruency. Indeed, a considerable number of previous studies have demonstrated that the dot probe task has poor internal and test-retest reliability for measuring the attentional bias to a threatening stimulus, based on reaction times evaluated in non-clinical populations, and have proposed that ERP would be a good indicator for performance in this task (Schmukle, 2005; Bar-Haim et al., 2007; Kappenman et al., 2014). However, we found a significant interaction between color and congruency. The difference in the result may be due to modification of the task, which may take into account the fact that the color red intensifies existing emotional attention priority. In addition, the reaction time for the color red was longer than that for the color green in both behavioral and ERP experiments. This may be because the color red captures the attention resources and interrupts the participants’ task-related attention, resulting in a longer reaction time for red stimuli.

We used ERPs to investigate whether there is a difference between attentional bias and emotional context information during the visual processing of the color red. First, using facial expressions as cues, we found that the color red leads to a larger P1 component than green. This result suggests that red may capture the early attention as it belongs to the long-wave colors and is associated with higher arousal. In fact, previous studies have found that even a simple red target circle elicits earlier latencies in the N2pc component than green targets (Fortier-Gauthier et al., 2013). In addition, a study has found that the color red increases blood pressure, skin electric potential, EEG alpha waves, and other physiological indicators (Ali, 1972; Jacobs and Hustmyer, 1974). Our results are in line with those of the aforementioned studies. Therefore, we propose that the color red captures initial attention, as indicated by the P1 amplitude in the dot probe task. Second, the interaction effect between color and congruency is significant. The color red, but not green, captured initial attention in the congruent condition with fearful, happy, and angry expressions, as shown by the P1 amplitude. The color red intensifies the attention during congruent conditions. This suggests that the attentional bias of the color red is influenced by the current tendency of the subjects. Indeed, behavioral studies have indicated that the color red intensifies attention to the stimuli that existing motivation tendency (Buechner et al., 2014, 2015). In our experiment, congruent conditional targets were considered the stimuli that existing attentional priority because emotional cues captured attention prior to the target presentation. Thus, red targets only intensify the emotional effect. Therefore, the red target modulates the P1 component, which takes into account the attentional bias toward red stimuli that is related to existing attentional priority.

The color red modulated the LPC, in addition to the P1 component, in response to angry, but not fearful and happy, facial expressions. Previous behavioral studies have found an association between the color red and anger conceptualization or experience in a discrete emotional context (Elliot and Aarts, 2011; Young et al., 2013). Our data suggest that differences in the amplitude of LPC between the red and green angry expression conditions may reflect the association between the color red and anger. The color red, thus, leads to higher arousal and attention priority, as shown by the LPC in the angry expression condition. It is noteworthy that there is no relationship between fearful expression and red based on the LPC amplitude. As mentioned above, the color red is often used to indicate dangerous situations. Elliot et al. (2007) has proposed that this association between the color red and danger often appears in the context of achievement, and undermines individuals’ performances in intellectual tasks. However, in emotional contexts, the color red not only facilitates the processing of angry expressions, but also enhances the processing of the concept of anger, although it does not facilitate the expression or conceptualization of fear (Fetterman et al., 2011; Young et al., 2013). Our results are consistent with the aforementioned findings, and rule out the idea that the color red has a generally negative emotional association. Thus, we provide additional evidence for a link between anger and the color red in a discrete emotional context. In addition to the association between the color red and anger, as shown by the LPC in our experiments, we found that, in the happy expression condition, the color green captured later attention, and led to a larger LPC than the color red. This result suggests that the green target, followed by the happy expression, sustains the perceiver’s attention, and reflects an association between the color green and happiness. In fact, previous studies also indicate that the color green is a pleasant hue in different tasks (Valdez and Mehrabian, 1994; Gil and Le Bigot, 2014). In general, LPC modulates red and green targets in the angry and happy expression conditions, respectively, which reflects the context-specific effects of colors on discrete emotions.

Taken together, the reaction time and P1 amplitude results indicate an attentional bias to the red target, which is congruent with emotional (anger, fear, and happiness) cues. The LPC amplitude reflects the emotional context-specific effects of color. In a discrete emotional context, individuals focused their attention unconsciously on the red and green targets, which followed the angry and happy expression cues, respectively. In our study, the context was represented by discrete emotional facial expressions. Our results suggest that the color red captures the initial attention in any motivational context (approach or withdrawal), and sustains the attention to the color if associated with a corresponding emotion. Our results may reconcile the difference between attentional bias and the context-specific effects of the color red, and highlight the need for investigators to study the mechanisms underlying the effect of the color red.

## Author Contributions

TX developed the study concept and performed the testing and data collection. TX and ZQ performed the data analysis and interpretation under the supervision of WL. TX drafted the manuscript. JS and MZ provided critical revisions. All authors contributed to the study design and approved the final version of the manuscript for submission.

## Conflict of Interest Statement

The authors declare that the research was conducted in the absence of any commercial or financial relationships that could be construed as a potential conflict of interest.
